# Gender differences in preferences, gateway effects, and potential motivations among e-cigarette users in China

**DOI:** 10.3389/fpubh.2025.1417218

**Published:** 2025-02-26

**Authors:** Wei Ji, Liyong Shi, Xinjun Lin, Zhiqiang Ji, Zhihuang Zhao, Yanping Chen, Pengxiang Huang, Xiali Wang, Xiaofang Dai, Jing Cheng, Lujun Guo, Diwei Wu, Yibiao Chen, Zhangcai Wu, Xiaoyang Chen

**Affiliations:** ^1^The Second Clinical College of Fujian Medical University, Quanzhou, Fujian, China; ^2^Department of Respiratory and Critical Care Medicine, The Second Affiliated Hospital of Fujian Medical University, Quanzhou, Fujian, China; ^3^Department of Graduate School, Zunyi Medical University, Zunyi, Guizhou, China; ^4^Quanzhou Medical College, Quanzhou, Fujian, China; ^5^Department of Orthopaedics, Dushu Lake Hospital Affiliated to Soochow University, Suzhou, Jiangsu, China; ^6^The Second Affiliated Hospital of Hainan Medical College, Haikou, Hainan, China; ^7^The First Affiliated Hospital of Fujian Medical University, Fuzhou, Fujian, China; ^8^FuZong Clinical Medical College of Fujian Medical University, Fuzhou, Fujian, China

**Keywords:** e-cigarettes, gender differences, preferences, gateway effect, addictive dependence, potential use motivation

## Abstract

**Objective:**

A cross-sectional survey was conducted to investigate the differences in preferences, gateway effects, and potential motivations for e-cigarette use among different genders of e-cigarette users in China, thereby providing ideas for the development of public prevention and intervention measures for e-cigarettes.

**Methods:**

This study adopted a combination of online web questionnaires and offline distribution questionnaires to survey 640 e-cigarette users by snowball sampling and convenience sampling in China in 2023. We used the Potential E-Cigarette Dependence Scale, the E-Cigarette Preference Scale, and the General Information Scale to conduct the survey and assessment, and surveyed 360 individuals of those who only used traditional cigarettes for comparison.

**Results:**

(1) The total number of participants in this research study was 1792, the mean age was 26.9 ± 9.0 years and the number of e-cigarette users was 640, of which 535 (83.6%) were males and 105 (16.4%) were females. (2) There was a statistically significant association between e-cigarette flavor preferences (flavor/nicotine) and gender (*p* < 0.05). Compared to males, females preferred e-cigarettes with scented/tobacco/fruit/beverage/nicotine (0 mg/12 mg) flavors. (3) Compared with males, females were more likely to “Transition to using traditional cigarettes after smoking e-cigarettes (Gateway effect)” (*p* < 0.05). (4) Whereas males were more likely to experience “Craving for traditional cigarettes after using e-cigarettes (Induction effect)” (*p* < 0.05). (5) There were significant gender differences in motivations for e-cigarette use. Males were more likely to use e-cigarettes to “quit traditional cigarettes,” whereas e-cigarette use was more closely related to self-perceptions of “feeling cool.” (6) Multi-factorial logistic regression analysis showed that there was a significant correlation between “e-cigarette liquid flavor (tobacco/fruit/beverage), e-cigarette liquid nicotine concentration (0 mg/12 mg)” and “Gender” (*p* < 0.05); Gateway effect was significantly correlated with “Gender and Age”(*p* < 0.05); E-cigarette addiction dependence was not significantly correlated with gender; Mild addiction to e-cigarettes was significantly correlated with “hope e-cigarettes carry nicotine, and the motivations for choosing e-cigarettes (to quit traditional cigarettes)” (*p* < 0.05). Severe addiction to e-cigarettes was significantly correlated with “hope e-cigarettes carry nicotine, and the motivations for choosing e-cigarettes (unable to use traditional cigarettes in public)” (*p* < 0.05).

**Conclusion:**

Among Chinese e-cigarette users, females preferred e-cigarettes with special flavors, and either without or with middling concentrations of nicotine. The gateway effect was more prominent in females and adolescents, and the induction effect was more notable in males. There was no significant correlation between addiction dependence on e-cigarettes and gender. E-cigarette use was more likely to be motivated by a desire to quit using traditional cigarettes in males, whereas women were more likely to be motivated by “self-perception.”

## 1 Introduction

Tobacco dependence remains a critical global public health challenge, directly contributing to over 7 million annual deaths worldwide (1, 2). Existing tobacco control strategies predominantly target smoking (3). Governments around the world are trying to find effective ways to curb smoking, with many turning to e-cigarettes (4, 5). The popularity of e-cigarettes is growing at an alarming rate (6). China alone has more than 10 million e-cigarette users (7). American adolescents e-cigarette use prevalence escalated 14-fold between 2011 and 2017 (1.5–20.8%) (8). New Zealand reports comparable trends, with adolescent usage rates surging from 7 to 20% within 2 years (9). Significant intergroup usage disparities persist, with gender-specific determinants remaining poorly characterized.

E-cigarettes are electronic products that mimic traditional cigarettes, producing an aerosol using a battery-powered atomizer that heats a liquid containing a variety of compounds including nicotine, additives, and flavorings (10, 11). Though there are a variety of e-cigarettes available on the market (Vape pens, Box Mod, refillable Pod Vape, etc.), all involve heating and atomizing an “e-liquid” (12). E-liquids are available in thousands of flavors, many designed to meet the preferences of youth. Flavoring may promote the initiation and continuation of e-cigarette use, and potentially pose an additional health risk to users (13). Australian youth predominantly select nicotine-containing or flavored e-cigarettes (13). Among the Chinese e-cigarette user group, there was a greater inclination toward e-cigarettes with fruit flavor (14).

The prevalence of e-cigarette use is rising, but there are still limitations in our understanding of its addictive potential, dependence, safety, usage motivations, and other aspects (15). What’s more, there appears to be a gateway effect (16). Some studies have shown that the use of e-cigarettes leads to up to four times the prevalence of traditional cigarette smoking in never smokers compared to the general population (17–19). E-cigarette use amongst adolescents and adults is rising, but addiction, dependence, safety and motivations around use are poorly understood (15). Some studies have shown that e-cigarette users are up to four times more likely to smoke traditional cigarettes than the general population (17–19), leading some authors to describe a “gateway effect” (16).

China represents a crucial focal point for studying the landscape of e-cigarettes, owing to its dual distinction as the country with the highest number of tobacco users globally and as the primary producer of the majority of e-cigarette products worldwide (14). It is important to evaluate and describe the preferences, extent of gateway effect, addiction dependence, and motivations of e-cigarette users in different gender groups to better develop public health interventions. The purpose of this article is to study the status of e-cigarette’s preferences and addiction dependence in different gender groups of e-cigarette users in China, to verify the existence of the gateway effect, and to explore the motivations for use, in order to elucidate the root causes of differences in gender groups of e-cigarette users, and to provide recommendations for the formulation of e-cigarette public health measures.

## 2 Methods

### 2.1 Study design and participants

In this study, snowball sampling and convenience sampling were conducted in 23 provinces and 4 municipalities in China from April to August 2023 through combined online and offline questionnaires (the study does not include the five autonomous regions and two special administrative regions within China). We used the Potential e-cigarette Dependence Scale (15) (Alpha coefficient is 0.862), the e-cigarette Preferences Scale (13), and the General Information Scale to conduct the survey and assessment. Questions include gender, age, height, weight, smoking history, recent tobacco use, order of use of e-cigarettes and traditional cigarettes, tobacco addiction and dependence, knowledge of e-cigarettes, and motivation for using e-cigarettes. A total of 2,213 questionnaires were distributed in this study and 1,792 valid questionnaires were recovered, with an effective recovery rate of 81.0%. Of the returned surveys, 1,228 participants were male and 564 were female. E-cigarette users accounted for 35.7% of the surveyed population (535 males and 105 females).

Before commencing the questionnaire survey, an additional investigation was conducted to delve deeper into the motivations behind e-cigarette usage among participants. The 60 e-cigarette users (38 males and 22 females) were interviewed to obtain open-ended responses regarding their reasons for using e-cigarettes. No predefined answers or options were provided, allowing participants to freely express their motivations. The motivations for their use of e-cigarettes were summarized by members of the team and ultimately included in the questionnaire. To avoid potential bias, the 60 e-cigarette users who participated in this preliminary investigation were not included in the subsequent questionnaire survey. These individuals were selected from 23 provinces and 4 municipalities in China (The study does not include the five autonomous regions and two special administrative regions within China), utilizing convenience and snowball sampling methods identical to those employed in the main survey.

All methods were performed in accordance with the relevant guidelines and regulations.

### 2.2 Sample size

The sample size was determined according to the formula ($n=\frac{\mu^{2}\alpha \pi \left(1-\pi \right)}{\delta^{2}}$) (20). *π* is the prediction rate of e-cigarette use. To minimize the error, we set π as 0.08 [Based on the 2020 Report on Health Hazards of Smoking in China (7)]. δ is the allowable error, the maximum error of the sample rate, and the overall rate that should be controlled range, we set the allowable error as 0.1π. We set *α* as 0.05, a confidence interval is 95%, and then the corresponding *μ* is 1.96. Using this method, we determine that n is 220 (e-cigarette users), and considering the 20% lost interview rate, 275 e-cigarette users need to be surveyed. To minimize error, there were 320 e-cigarette users in this study.

### 2.3 Tobacco use definition

We defined current traditional cigarette users as those who had smoked more than 100 conventional cigarettes in a lifetime, and who had smoked at least one conventional cigarette in the past month (21). E-cigarette users were defined as those who had used e-cigarettes in the last month and vaped more than once per day. Dual users needed to meet both criteria. Other users were excluded from analysis.

### 2.4 Potential e-cigarette dependence scale

The Potential e-cigarette Dependence Scale (15) was developed by the American Tobacco Center for Regulatory Science (TCORS) Measurement Workgroup and a 10-person external subject-matter expert panel after reviewing a selection of 12 currently used tobacco dependence measures. The Potential e-Cigarette Dependence Scale consists of 10 entries: (1) Quantity and frequency of use, (2) Tolerance, (3) Perceived benefits, (4) Withdrawal symptoms, (5) Craving/urge to use, (6) Use despite harm, (7) Impaired control, (8) Automaticity, (9) Preferred over competing rewards, and (10) Sensory dependence. However, TCORS does not propose a definitive grading for the E-Cigarette Dependence Scale. We were basing our grading of tobacco dependence addiction on the Diagnostic and Statistical Manual of Mental Disorders Fifth Edition (DSM-5). One point is awarded for each item met, 0–1 points for no apparent tobacco addiction, 2–3 points for mild tobacco dependence addiction, 4–5 points for moderate tobacco dependence addiction, and 6 or more points for severe tobacco dependence addiction. To avoid selection bias in assessment methods, we also chose the heaviness of smoking index (HSI) (22) for validation of tobacco dependence addiction, and the HSI score ≥ 4 was considered severe tobacco addiction.

### 2.5 E-cigarette preferences scale

The E-cigarette Preferences Scale (12) focuses on the preference of e-cigarette choices for those who use e-cigarettes who participated in the survey. Example questions include: “Do you prefer to choose e-cigarettes with scented flavors?” “Do you prefer e-cigarettes with fruit, tobacco, beverage, or herbal flavors?” Data on nicotine-containing e-cigarette use is also sampled, including the amount of nicotine in the e-liquid. We have designated 0 mg/L as “no nicotine,” 6 mg/L “low concentration,” 12 mg/L “moderate concentration,” and > 18 mg/L as “high concentration.”

### 2.6 General information scale

The General Information Scale included basic information about the respondents such as gender, age, weight, and height. Respondents were asked about their smoking history, including the amount, frequency and duration of use in years. If there was a history of both traditional and e-cigarette use, respondents were asked which modality was used first, as well as their motivations for e-cigarette use.

### 2.7 Data analysis

This study used SPSS 27.0 (copyright license) software to analyze the data. Independent samples t-test and chi-square test were performed on the data by spss27.0 to initially clarify whether the differences between the variables were statistically significant. We took the likelihood ratio test, estimated values as parameters, took the confidence interval as 95%, and determined the optimal model through goodness-of-fit, pseudo-R-square, and simulated fitting criteria, after several attempts of main effects and interaction effects. For variables with statistical significance, we performed logistic regression analysis through the optimization model. All statistical tests were two-tailed (*α* = 0.05).

### 2.8 Questionnaire quality assurance

To ensure the quality of questionnaire completion, we conducted logical relationship validity tests, by designing some of the questions with mutually exclusive or inclusive options and screened out questionnaires with inconsistent responses on these questions. To ensure that the samples were real and unique, each IP address and cell phone number could only fill out the questionnaire once. We conducted manual screening after the questionnaires were collected, and the responses were invalidated for garbled and negative responses (garbled and negative responses pertain to answers that exhibit clear inconsistencies or physiologically implausible statements. Examples include selecting both “smoked e-cigarettes after smoking traditional cigarettes” and “smoked traditional cigarettes after smoking e-cigarettes, “or claiming to have smoked 1,000 traditional cigarettes in a single day).

## 3 Results

### 3.1 Characteristics of participants — gender distribution

The total number of participants in this research study was 1792. There were 1,228 (68.5%) males and 564 (31.4%) females, with an average age of 26.9 ± 9.0 years. Specific details are shown in Table 1.

**Table 1:** Characteristics of participants.

	Participants, *n* (%)	
	Males	Females
All participants	**1,228 (68.5)**	**564 (31.5)**
Age		
<18	41 (3.3)	60 (10.7)
≥18	1,187 (96.7)	504 (89.3)
E-cigarette smoking	**535 (83.5)**	**105 (16.4)**
E-cigarette addiction dependence scores		
0–1	391 (73.1)	75 (71.4)
2–3	54 (10.1)	8 (7.6)
4–5	36 (6.7)	12 (11.5)
≥6	54 (10.1)	10 (9.5)
Craving for traditional cigarettes after inhaling e-cigarettes (Induction Effect)		
Yes	259 (48.1)	0 (0)
No	276 (51.5)	105 (100.0)
Traditional cigarette smoking	**580 (87.9)**	**80 (12.1)**
HSI		
<4	454 (78.3)	79 (98.8)
≥4	126 (21.7)	1 (1.2)
Smoking both traditional cigarettes and e-cigarettes	**520 (86.7)**	**80 (13.3)**
Started smoking traditional cigarettes after smoking e-cigarettes (Gateway Effect)		
Yes	60 (11.5)	20 (25.0)
No	460 (88.5)	60 (75.0)

The table provides an overview of participant characteristics and gender distribution across different groups, including all participants, e-cigarette users, traditional cigarette users, and individuals who use both traditional cigarettes and e-cigarettes. Bold text in the table denotes the respective sample population, while bolded values indicate the total number and percentage of participants within each sample group. The values listed below represent the corresponding structural composition and percentage. HSI: The heaviness of smoking index.

### 3.2 Preferences for use — gender differences

**Figure 1:**
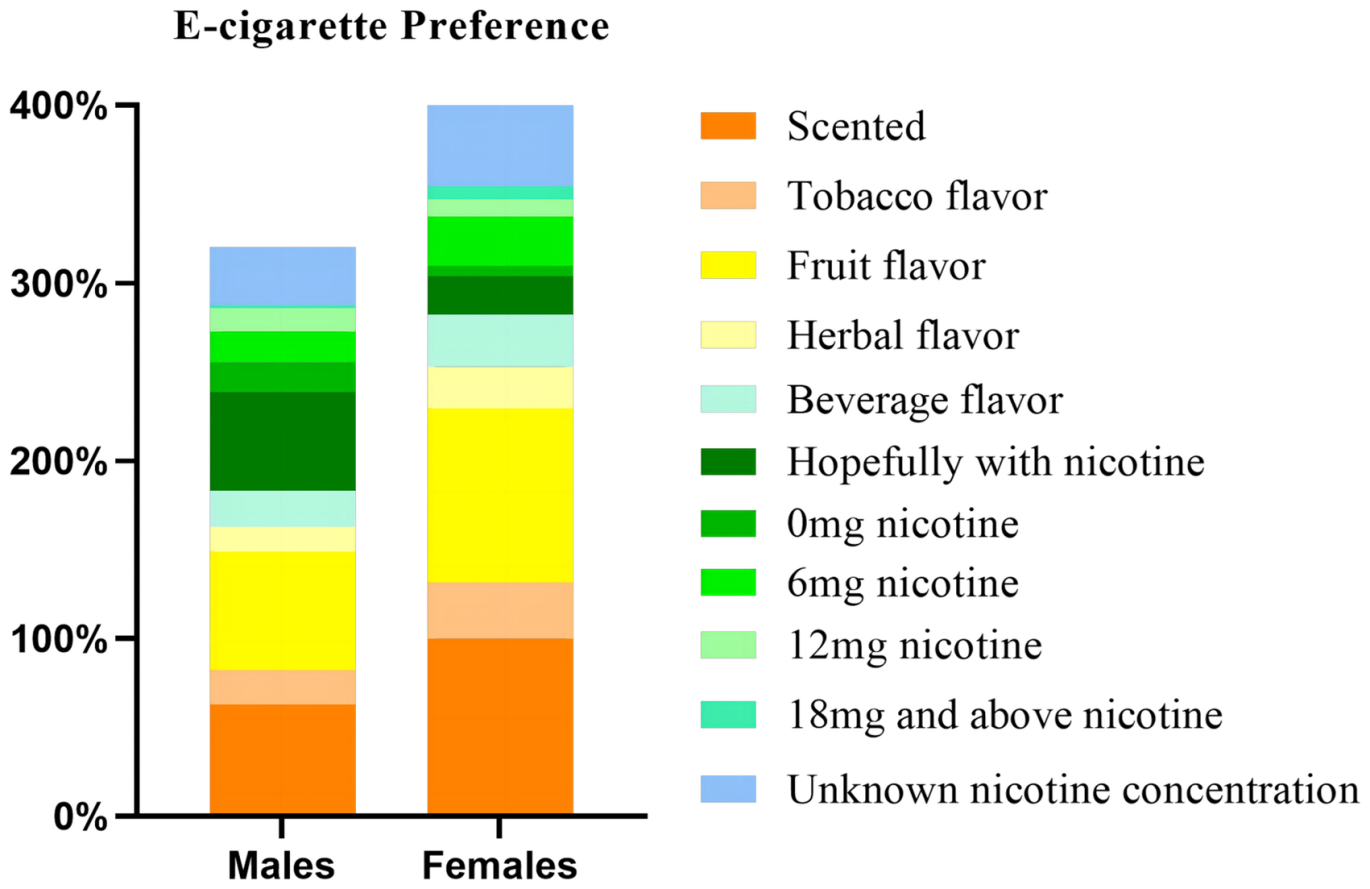
Preferences of e-cigarette users — gender comparison. This figure presents a gender-based comparison of e-cigarette preference among different groups of e-cigarette users, expressed as percentages. The graph shows the variations between males and females regarding different flavors of e-cigarettes. The vertical axis of the graph represents the percentage, while the horizontal axis represents gender.

We performed multi-factorial unconditional logistic regression analyses with “Gender” as the independent variable and “e-liquid (flavor/nicotine concentration)” as the dependent variable (Inclusion criterion was *p* < 0.05, exclusion criterion was *p* > 0.10). The results of the analysis showed that there was a significant correlation between “e-cigarette liquid flavor (tobacco/fruit/drink), e-cigarette liquid nicotine concentration (0 mg/12 mg)” and “Gender.” “e-cigarette liquid flavor (herbal), e-cigarette liquid nicotine concentration (6 mg/18 mg and above/unknown concentration)” had no significant correlation with “Gender.” In addition, among the 640 e-cigarette participants, all female users (105) answered positively on e-cigarette liquid flavor (scent). Details are shown in Table 2. The rows with *β* of 0 and OR of 1 in Table 2 were used as references for this variable. Figure 1 shows the composition ratio of e-cigarette use preferences among participants.

**Table 2:** E-cigarette preferences.

Variables							
	Males, *n* (%)	Females, *n* (%)	*T*	*p* ^a^	*χ*2	OR (95%CI)	*p* ^b^
Prefer scented e-cigarettes			12.593	**<0.001**	27.577	/	**<0.001**
Yes	335 (62.6)	105 (100)					
No	200 (37.4)	0 (0)					
Hope e-cigarettes carry nicotine			−4.557	**<0.001**	19.617	4.515 (2.221–9.177)	**<0.001**
Yes	296 (55.3)	22 (21.0)					
No	239 (44.7)	83 (79.0)					

Multi-factor logistic regression analysis					
		*β*	Wald χ^2^	OR (95%CI)	*p* ^c^
E-liquid flavor preferences					
Tobacco flavors	Males	−0.589	4.106	0.555 (0.314–0.981)	**0.043**
	Females	0		1	
Fruit flavors	Males	−0.745	19.099	0.475 (0.340–0.663)	**<0.001**
	Females	0		1	
Herbal flavors	Males	−0.508	2.274	0.602 (0.311–1.164)	0.132
	Females	0		1	
Beverage flavors	Males	−0.695	5.488	0.499 (0.279–0.893)	**0.019**
	Females	0		1	
E-cigarette nicotine concentration					
None, 0 mg	Males	−2.14	12.783	0.118 (0.036–0.380)	**<0.001**
	Females	0		1	
Low, 6 mg	Males	−0.577	3.461	0.561 (0.306–1.031)	0.063
	Females	0		1	
Middle, 12 mg	Males	−1.34	7.747	0.262 (0.102–0.673)	**0.005**
	Females	0		1	
High, 18 mg	Males	0.413	0.377	1.511 (0.404–5.653)	0.539
	Females	0		1	
Unknown nicotine concentration	Males	−0.397	3.423	0.673 (0.442–1.024)	0.064
	Females	0		1	

The primary focus of this table pertains to e-cigarette preferences, including factors such as e-cigarette flavor, scent presence, nicotine concentration, and other relevant aspects. The table incorporates relevant statistical analyses, including t-tests, chi-square tests, and multi-factor logistic regression analysis. Statistically significant values within the table are indicated in bold.

### 3.3 E-cigarette gateway effect and induction effect — gender differences

We performed multi-factorial unconditional logistic regression analyses with “Gender and Age” as independent variables and “Starting to smoke traditional cigarettes after using e-cigarettes” as a dependent variable (Inclusion criterion was *p* < 0.05, exclusion criterion was *p* > 0.10). Since the HSI is used to assess the degree of addiction to traditional cigarettes, there is a sequential relationship between the variable “starting to smoke traditional cigarettes after using e-cigarettes (Gateway effect)” and the HSI in the study. Hence, we did not conduct a logistic regression analysis on it. At the same time, we took “Craving for traditional cigarettes after inhaling e-cigarettes (Induction effect)” as the dependent variable and “Gender, Age, and HSI” as the independent variables, and then performed multi-factorial unconditional logistic regression analyses (the inclusion criterion was *p* < 0.05). The rows with *β* of 0 and OR of 1 in Table 3 were used as references for this variable.

**Table 3:** E-cigarette gateway effect and induction effect.

Variables							
	Males, *n* (%)	Females, *n* (%)	*T*	*p* ^a^	χ2	OR (95%CI)	*p* ^b^
Starting to smoke traditional cigarettes after using e-cigarettes (Gateway effect)							
Yes	60 (10)	20 (3.3)	2.345	**<0.001**	5.436	0.391 (0.174–0.88)	**0.02**
No	460 (76.6)	60 (10)					
Craving for traditional cigarettes after using e-cigarettes (Induction effect)			−6.885	**<0.001**	41.51	/	**<0.001**
Yes	259 (40.5)	0 (0)					
No	276 (43.1)	105 (16.4)					

Multi-factor logistic regression analysis							
		*β*		Wald χ2	OR (95%CI)		*p* ^c^
Starting to smoke traditional cigarettes after using e-cigarettes (Gateway effect)							
	Gender	Males	−0.904	4.123	0.405 (0.169–0.969)		**0.042**
		Females	0		1		
	Age	<18	2.462	23.161	11.728 (4.303–31.966)		**<0.001**
		≥18	0		1		
Craving for traditional cigarettes after using e-cigarettes (Induction effect)							
	Gender	Males	/	/	/	/	
		Females					
	Age	<18	0.152	0.065	1.164 (0.361–3.759)		0.799
		≥18	0		1		
	HSI	<4	−2.269	46.363	0.103 (0.054–0.199)		**<0.001**
		≥4	0		1		

The table provides a summary of the gateway and induction effects associated with e-cigarettes, including corresponding t-tests, chi-square tests, and multi-factor logistic regression analysis. The gateway effect reflects the occurrence of event B as a result of event A (event B had not occurred previously). The induction effect measures the extent to which event A exacerbates event B (event B already existed before event A). Statistically significant values are indicated in bold within the table. HSI: The heaviness of smoking index.

**Figure 2:**
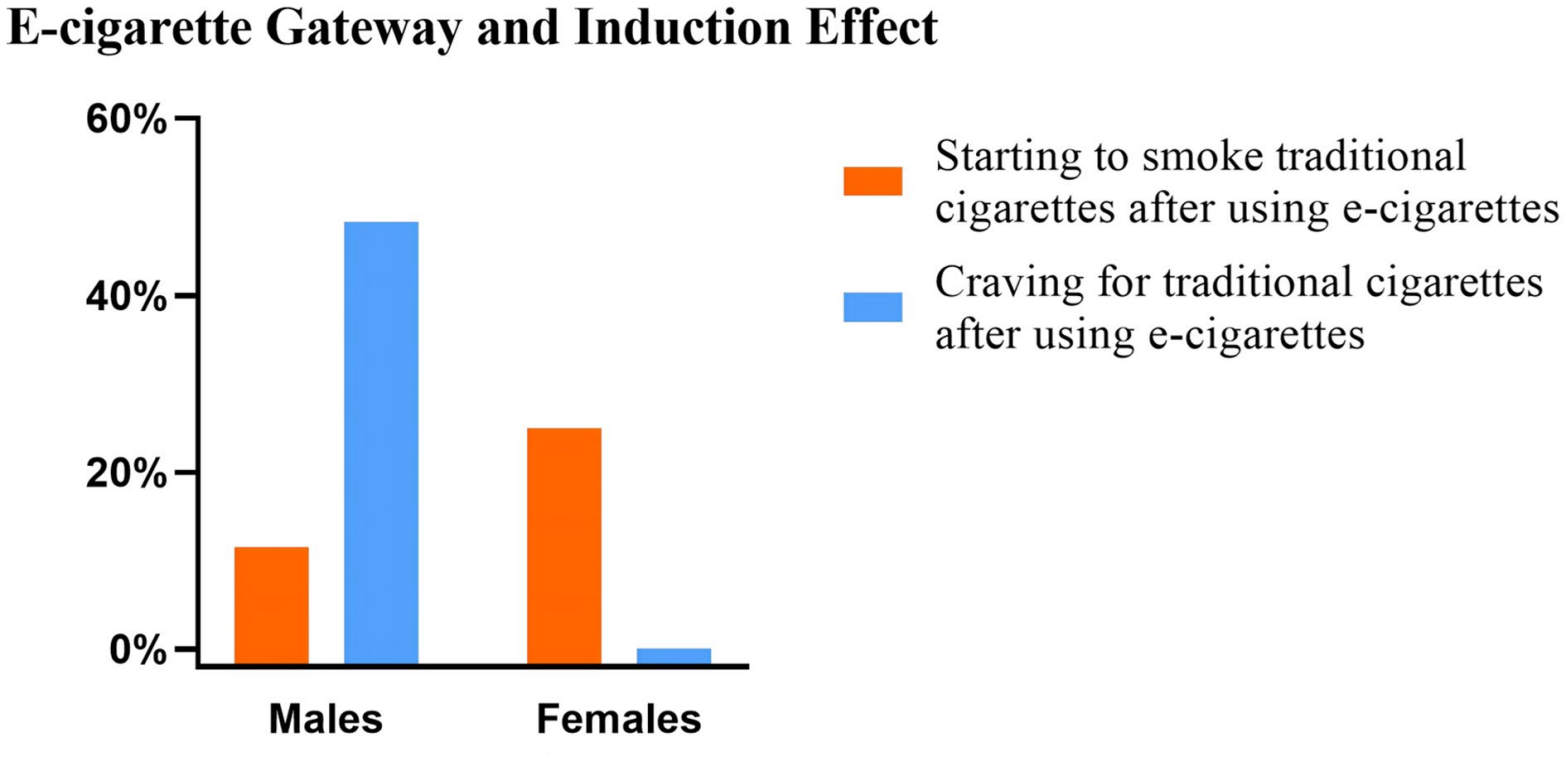
Gateway Effect and Induction Effect of e-cigarette users — gender comparison. The graphical representation presented the comparison of e-cigarette gateway and induction effects between male and female groups. The vertical axis of the graph represents the percentage, while the horizontal axis corresponds to gender.

The results of the analysis showed that there was a significant correlation between “Starting to smoke traditional cigarettes after using e-cigarettes (Gateway effect)” and “Gender and Age.” “Craving for traditional cigarettes after using e-cigarettes (Induction effect)” was significantly correlated with “HSI,” but the induction effect had no significant correlation with “Age “. It should be noted that all 105 women who used e-cigarettes in the participants chose a negative answer on the induction effect. The details are shown in Table 3 and Figure 2.

### 3.4 E-cigarette dependence addiction — gender differences

To comprehensively investigate the underlying factors contributing to varying degrees of e-cigarette dependence and addiction, we incorporated participants’ motivations for using e-cigarettes, gathered during the previous pre-survey, into the construction of the multi-factor logistic regression model.

We conducted multi-factorial unconditional logistic regression analyses with “gender, hope e-cigarettes carry nicotine, and reason for choosing e-cigarettes (unable to use traditional cigarettes in public/attempt to quit traditional cigarettes)” as the independent variables, and “degree of dependence and addiction to e-cigarettes” as the dependent variable (Inclusion criteria *p* < 0.05, exclusion criteria *p* > 0.10). The rows with *β* of 0 and OR of 1 in Table 4 were used as references for this variable.

**Table 4:** Multi-factor logistic regression analysis—E-cigarette dependence addiction.

	Factors	Variables	*β*	Wald χ^2^	OR (95%CI)	*p*
Mild addiction, 2–3 points						
	Gender	Males	0.21	0.138	1.234 (0.407–3.737)	0.711
		Females	0		1	
	Hope e-cigarettes carry nicotine	Yes	1.488	7.254	4.429 (1.500–13.081)	**0.007**
		No	0		1	
	To quit traditional cigarettes	Yes	−1.198	5.688	0.302 (0.113–0.808)	**0.017**
		No	0		1	
	Unable to use traditional cigarettes in public	Yes	0.038	0.007	1.039 (0.414–2.610)	0.935
		No	0		1	
Moderate addiction, 4–5points						
	Gender	Males	−0.601	16.511	0.548 (0.204–1.475)	0.234
		Females	0		1	
	Hope e-cigarettes carry nicotine	Yes	0.153	0.106	1.165 (0.712–5.204)	0.744
		No	0		1	
	To quit traditional cigarettes	Yes	0.327	0.507	1.387 (0.564–3.410)	0.477
		No	0		1	
	Unable to use traditional cigarettes in public	Yes	−0.388	0.651	0.679 (0.265–1.740)	0.42
		No	0		1	
Severe addiction, 6 points and above						
	Gender	Males	−0.013	17.109	0.987 (0.356–2.732)	0.98
		Females	0		1	
	Hope e-cigarettes carry nicotine	Yes	−1.031	5.438	0.775 (0.252–2.390)	**0.02**
		No	0		1	
	To quit traditional cigarettes	Yes	0.045	0.012	1.046 (0476–2.301)	0.911
		No	0		1	
	Unable to use traditional cigarettes in public	Yes	1.054	4.105	2.868 (1.035–7.950)	**0.043**
		No	0		1	

This table shows the multi-factor logistic regression analysis of e-cigarette dependence addiction on different degrees of severity. The factors influencing mild addiction, moderate addiction, and severe addiction are listed in order, with no apparent addiction serving as a reference. The independent variables in the model are gender and partial motivation for e-cigarette use. Statistically significant values are indicated in bold within the table.

**Figure 3:**
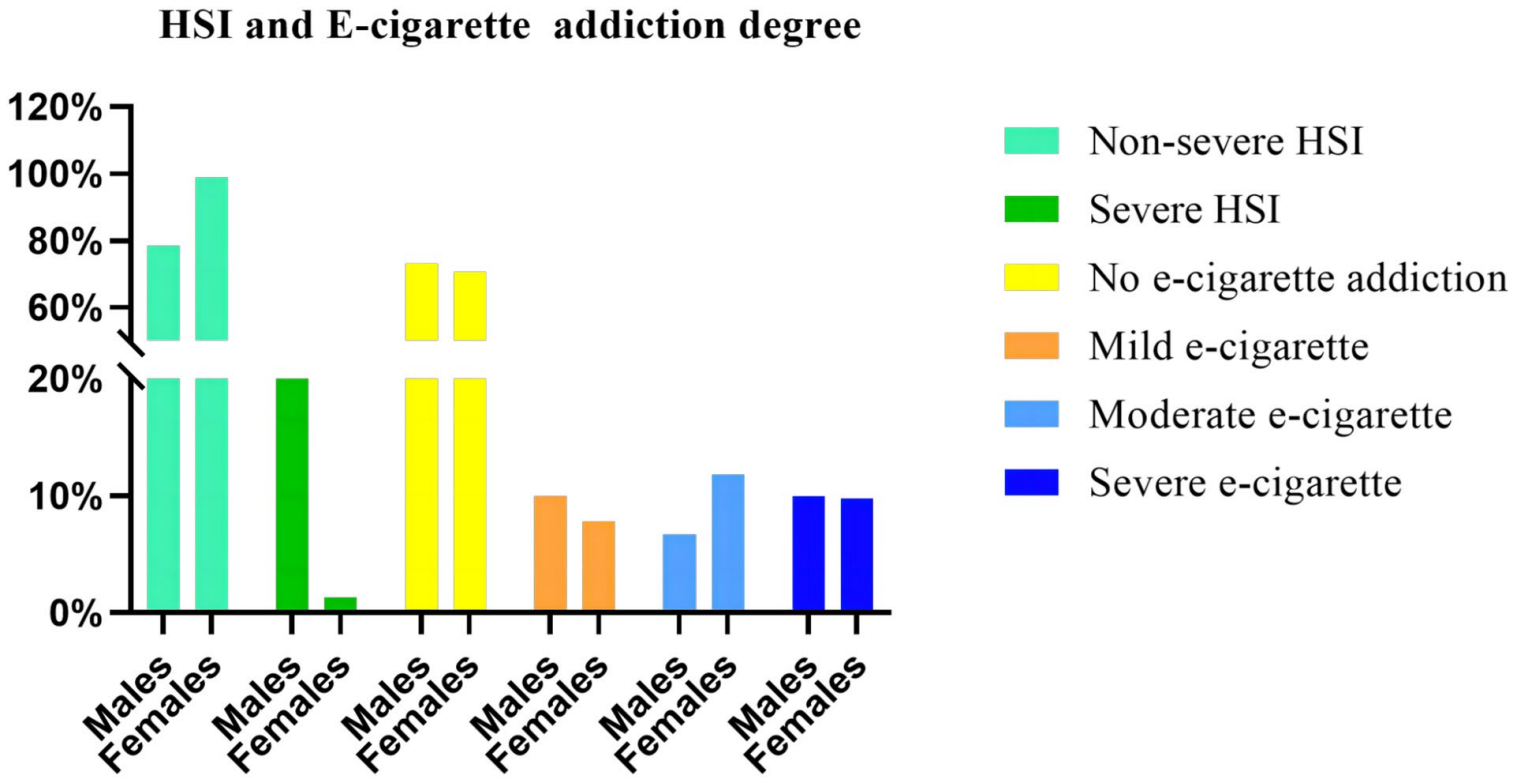
HSI and e-cigarette addiction degree — gender comparison. This figure presents a comparison of the addiction degrees between traditional cigarettes and e-cigarettes within the male and female groups. The vertical axis of the graph represents the percentage, while the horizontal axis corresponds to gender. The truncated slashes in the vertical coordinates refer to split points of 20 and 50%. HSI: the heaviness of smoking index.

**Figure 4:**
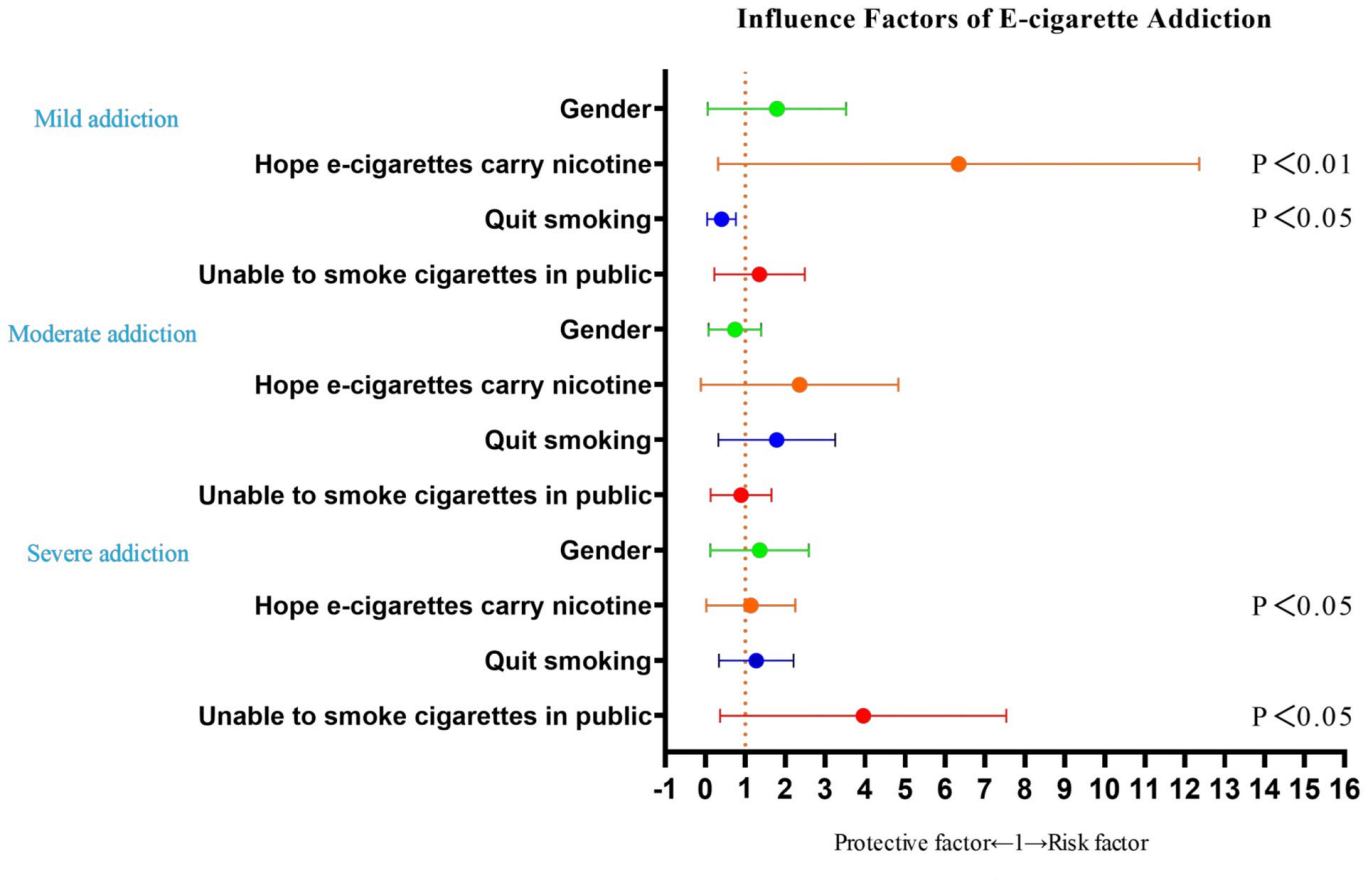
This figure displays the odds ratios (OR) of factors influencing mild, moderate, and severe e-cigarette addiction, arranged from top to bottom. The orange dotted line in the figure represents the reference point of 1, distinguishing between protective factors and risk factors. Values >1 indicate risk factors, while values <1 indicate protective factors. The *p*-values labeled individually within the figure correspond to variables that demonstrate statistical significance relative to the vertical axis.

The results of the analysis showed that based on “no addiction to e-cigarettes (0–1 points)” as a control, mild addiction to e-cigarettes was significantly correlated with “hope e-cigarettes carry nicotine, and the reason for choosing e-cigarettes (to quit traditional cigarettes),” and was not significantly correlated with “gender, and the reason for choosing e-cigarettes (unable to use traditional cigarettes in public)”; Moderate addiction to e-cigarettes was not significantly associated with “gender, hope e-cigarettes carry nicotine, and reason for choosing e-cigarettes (unable to use traditional cigarettes in public/attempt to quit traditional cigarettes)”; Severe addiction to e-cigarettes was significantly associated with “hope e-cigarettes carry nicotine, and the reason for choosing e-cigarettes (unable to use traditional cigarettes in public),” and not significantly associated with “gender, and the reason for choosing e-cigarettes (to quit traditional cigarettes).” Details can be found in Table 4 and Figures 3, 4.

### 3.5 Potential motivations for e-cigarette use — gender differences

In order to further explore the potential motivations for choosing e-cigarettes in the e-cigarette-using group, 60 e-cigarette users were asked and their motivations for using e-cigarettes were included in the questionnaire before the questionnaire survey was formally started (These 60 e-cigarette users did not participate in the questionnaire response to avoid bias).

**Figure 5:**
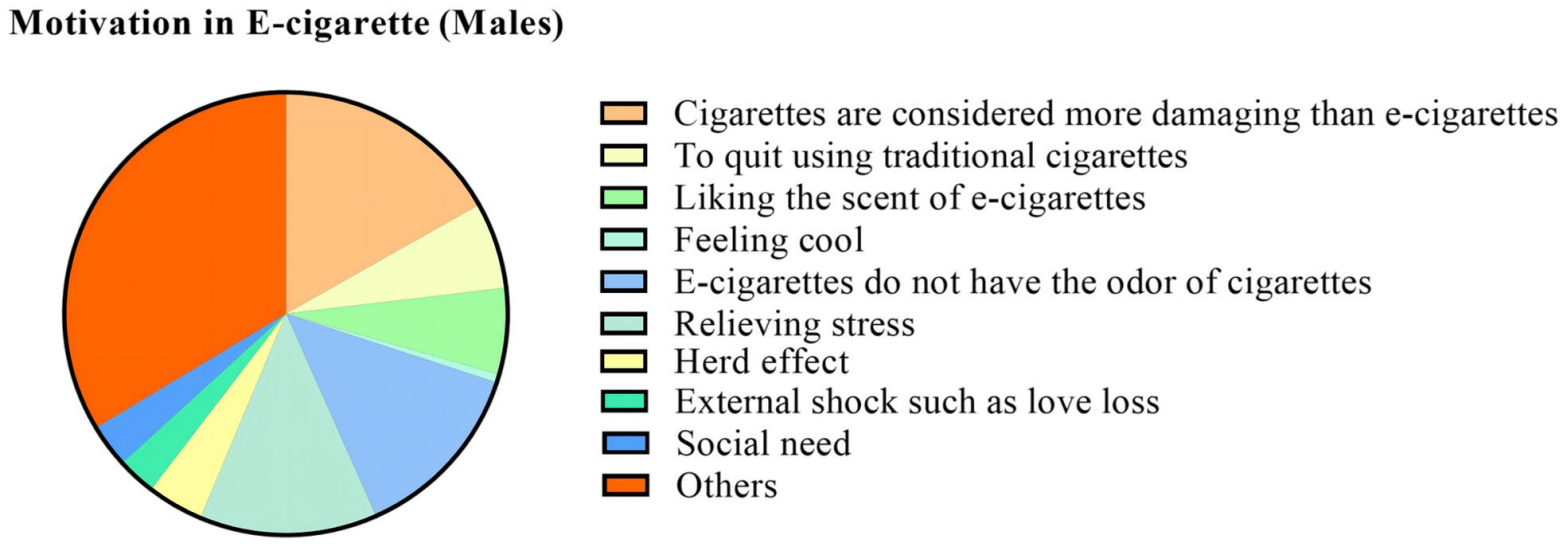
Motivation in e-cigarette (males). The proportions in the graph are the proportion of choices in the number of men in the group.

**Figure 6:**
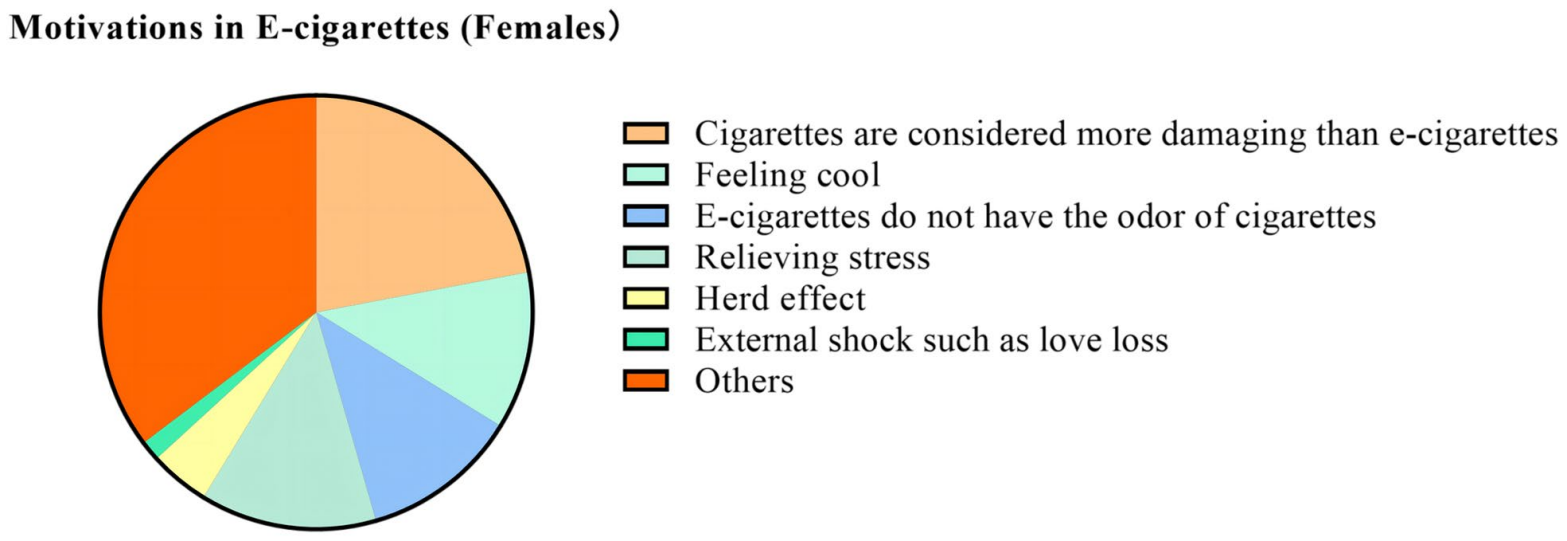
Motivation in e-cigarette (females). The proportions in the graph are the proportion of choices in the number of women in the group.

We found that “the belief that traditional cigarettes are more damaging than e-cigarettes, the belief that e-cigarettes can relieve stress, and the belief that e-cigarettes do not have the odor of cigarettes” are the main motivations for e-cigarette use. In addition, “to quit using traditional cigarettes and to like the scent of e-cigarettes” are unique motivations for the male group. However, among female users, the self-perception of “feeling cool” is a prominent motivation for use. In addition, “social need, herd effect (Surrounded by friends or family who smoke and follow), and external shock such as love loss” is one of the important motivations for choosing to use e-cigarettes. Details are shown in Table 5 and Figures 5, 6.

**Table 5:** Motivations for e-cigarette use.

Factors	Variables	Males, *n* (538, %)	Females, *n* (102, %)	χ2 (df)	*p*-value
Motivations				40.613 (10)	**<0.001**
	Cigarettes are considered more damaging than e-cigarettes	105 (19.6)	31 (29.5)		
	To quit using traditional cigarettes	40 (7.4)	0 (0.0)		
	Liking the scent of e-cigarettes	40 (7.4)	0 (0.0)		
	Feeling cool	4 (0.7)	16 (15.7)		
	E-cigarettes do not have the odor of cigarettes	84 (15.6)	16 (15.7)		
	Relieving stress	82 (15.2)	18 (17.6)		
	Surrounded by friends or family who smoke and follow (herd effect)	26 (4.8)	12 (11.8)		
	External shock such as love loss	18 (3.3)	2 (2.0)		
	Social need	20 (3.7)	0 (0.0)		
	Others	210 (39.3)	50 (47.6)		

This table shows the chi-square analysis of motivation to use e-cigarettes in different gender groups. Statistically significant values are indicated in bold within the table.

## 4 Discussion

Smoking is a well-documented risk factor for adverse health outcomes. Traditional cigarettes impose substantial burdens on global public health, socioeconomic systems, and individual well-being. Although there is not enough evidence to clarify the harmful effects of e-cigarettes (21), some studies have indicated that e-cigarettes confer a certain degree of damage to the human body (23, 24). According to the 2020 Report on Health Hazards of Smoking in China, the current prevalence of e-cigarettes is 0.9% nationally (7). Our survey identified 640 e-cigarette users (mean age 26.9 ± 9.0 years). The predominance of young users suggests sustained growth of e-cigarette adoption in China. As the global leader in tobacco production and consumption, China faces projected annual tobacco-attributable deaths of 2 million by 2030 without effective interventions (25), posing substantial societal burdens domestically and globally. Therefore, effective interventions targeting both conventional and electronic tobacco products are imperative. At present, the proportion of male users of traditional cigarettes in China is about 96.1%, and the proportion of female users is about 3.9% (7), but there is no specific statistical data on the utilization rate of e-cigarettes by gender in the Chinese e-cigarette user group. Our study reveals a gender disparity among Chinese e-cigarette users (83.6% male vs. 16.4% female), and the obvious difference in usage rates between males and females provides clues for exploring the characteristics of e-cigarette users. This contrasts with American patterns (51.5% male vs. 48.5% female users) (26), demonstrating cross-national gender variation. The reasons behind this phenomenon are unclear, which may be related to cultural practices, family attitudes, education, religious beliefs, or occupations.

The e-cigarette gateway effect refers to users who had not previously smoked traditional cigarettes beginning to smoke traditional cigarettes after smoking e-cigarettes (27). While emerging evidence supports the existence of this phenomenon (28), reproducibility challenges persist (29). Our study suggests there may be some degree of gateway effect amongst e-cigarette users, which is most prominent in females. Though the reasons behind this phenomenon are not clear, we hypothesize that it may be related to the following: (1) Males may have been exposed to traditional cigarettes in adolescence; (2) Differences in socialization between males and females; (3) Greater avoidance of health harmful activity amongst females (30). It is important to note that the presence of gateway effects in this study cannot exclude the possibility of “common liability” as a potential contributing factor (31).

We also found that the gateway effect was significantly stronger in adolescents than adults. It has been suggested that increased e-cigarette use among youth may represent a gateway to tobacco product use (29). This effect manifests as nicotine-naïve minors initiating e-cigarette use at higher rates, subsequently transitioning to combustible tobacco products. The European Tobacco Products Directive has suggested that the increase in the consumption of electronic cigarettes among young people may represent a gateway to the consumption of tobacco products (29). We think that this may be due to the fact that adolescents are in a period of sensitivity and curiosity to the outside world, and on this basis, a series of effects brought by contact with things will be more obvious. In addition, adolescents may perceive potential benefits associated with continued use of traditional cigarettes, including expedited nicotine delivery to the brain, leading to enhanced pleasurable effects or quicker alleviation of cravings (32).

We found that e-cigarettes seem to have a degree of induction effect (craving for traditional cigarettes after smoking e-cigarettes), which has not been mentioned in previous literature. Though many people choose to use e-cigarettes to quit smoking, we found its utility to be limited, and the induction effect may be associated with reverse promotion. This phenomenon exhibited male predominance without significant female correlation. This may be related to the higher degree of heavy traditional cigarette use among males, where tobacco addiction could be a more significant feature. However, the influence of various factors such as social, family, and personal factors cannot be ruled out, and the deeper reasons have yet to be further explored.

Perhaps owing to the rapid growth in the number e-cigarette users, there is little industry standardization of flavors, additives and sales. Out of concern for the potential dangers, the American Food & Drug Administration (FDA) has not yet approved the sale of non-tobacco flavored e-cigarettes and has called for more stringent regulations regarding the marketing of e-cigarette products. Likewise, the Australian government has imposed stricter regulations on e-cigarettes (33). In 2022, China’s state Tobacco Monopoly Administration published the *Measures for the Administration of Electronic Cigarettes*, which has banned the sale of non-tobacco flavored e-cigarettes and comprehensively strengthened regulation of the industry (34). This study identifies flavor appeal as a distinctive driver of e-cigarette adoption among Chinese male users, who constitute the predominant user demographic. Implementation of flavor restrictions excluding non-tobacco variants may serve to curb usage prevalence by targeting this population-specific motivator. Concurrently, reinforced regulatory oversight of manufacturing and distribution networks elevates procurement barriers, potentially suppressing consumption rates through controlled market accessibility. Previous studies have shown a preference for scented and nicotine-containing e-cigarettes amongst users (13), though these were limited to the adolescent group, and did not explore gender associations. Our study suggests that users of both genders prefer scented e-cigarette liquids, and that females prefer special flavors such as fruits, beverages and tobacco – though the root cause of this phenomenon is not clear. There are some studies which suggest e-cigarettes with sweet flavorings (such as fruit and beverages), can motivate people to continue to use e-cigarettes (35). We hypothesize that younger female users may be more sensitive to odors that produce pleasurable effects (35, 36).

Many countries have been gradually trying to implement e-cigarette control measures to curb e-cigarette use. Some scholars have suggested that the implementation of flavor bans may prevent youth from becoming dependent on tobacco, and thus less likely to use traditional cigarettes (37). There are studies which have shown that flavored e-cigarette products exacerbate the risk of addiction, with scent being a major driver behind usage (19, 27). It is worrying that in the face of the flavor ban in the United States, the smuggling and black-market trade of these products has become rampant, with many sales occurring through unofficial channels. Unregulated distribution channels increase exposure to substandard products with elevated health risks (37, 38). This may be because these restrictions have not removed the root incentive to smoke in e-cigarette users.

When we explored the motivations for using e-cigarettes, we found that “smoking cessation, stress relief, herd effect, social need, external shock, preference, self-perception” are important motivations for smokers to choose e-cigarettes. We believe that effective strategies to combat e-cigarette use will need to combine large-scale social awareness campaigns with psychological relief and social support. Government policy priorities should be to both reduce the harm to smokers (both psychological and physical) (39), to protect the health of non-smokers, and to address the motivations at the root of cigarette use. These motivations seem to differ between genders, with females more strongly motivated by stress relief, self-perception and the herd effect. These motivations may be more amenable to psychological relief and social support. Males, in contrast, are more heavily motivated by a desire to quit smoking traditional cigarettes, and to avoid the scent of traditional cigarettes. Those with severe tobacco dependence were also motivated to use e-cigarettes as they were not “able to smoke traditional cigarettes in public.” In this group, strategies such as a public smoke-free policy (including e-cigarettes) may be an effective solution. Amongst mild users who are more scent motivated, the current ban on flavors may be effective, as well as increasing awareness and availability of other smoking cessation products and strategies which are less harmful (40–42).

We found that there was a clear gateway effect among Chinese e-cigarette users, which is more prominent in females and adolescents. The majority of male e-cigarette users are dual users (both traditional and e-cigarettes), whereas female users tended to use e-cigarettes alone. We found that flavored e-liquids were preferred, with fruit, beverage, tobacco and scent flavorings to be the most popular amongst females, who also preferred no or moderate concentration nicotine products. We found e-cigarettes users to have less features of tobacco dependence, but to exhibit dependence regardless. Mild tobacco dependence was associated with a motivation to quit traditional cigarette smoking, but severe dependence was correlated with a desire to smoke in public where traditional cigarettes are banned. Both populations are more likely to use nicotine-containing e-liquids. In the exploration of potential motivations for e-cigarette use, we found that “cigarettes are considered more damaging than e-cigarettes, e-cigarettes do not have the odor of cigarettes, e-cigarette can relieve stress” are some of the main reasons why people choose to use e-cigarettes. In contrast, “to quit traditional cigarettes and to like the scent of e-cigarettes” were prominent in the male group, self-perceptions of “feeling cool” was more prominent in the female group. In addition, the “herd effect, social need, and external shock” are also potential motivations for use that cannot be ignored.

In conclusion, our findings regarding gender-specific distinctions in usage motivations, preference patterns, induction effects, and gateway effects among Chinese e-cigarette users warrant targeted public health interventions. Current evidence underscores the addictive potential of e-cigarettes and their documented adverse health impacts, necessitating prioritized preventive measures for non-user populations. The accelerating adoption rates of e-cigarettes further mandate urgent public education campaigns to address critical gaps in risk perception. Concurrently, regulatory reinforcement should focus on implementing real-time facial recognition systems for online sales platforms to ensure strict age verification, thereby mitigating underage access.

### 4.1 Strengths and limitations

This is the first study to investigate the differences in preferences, gateway effects, induction effects, addictive dependence, and potential motivations for use among different genders of e-cigarette users in China. We have explored the different characteristics of use among gender using a large-scale survey.

Some of the potential limitations of this study are worth discussing. First of all, due to the cross-sectional character of the study, the causality of the relationships observed in this study could not be determined. Second, the absence of objective measures of use preference and addictive dependence in the study may result in misclassification and recall bias. Thirdly, our survey included only 23 provinces and 4 municipalities in China, not including the other 5 autonomous regions and 2 special administrative regions within China. Fourth, we used convenience and snowball sampling methods. As a result, our study can only provide insights into the characteristics that may exist among a certain extent of the e-cigarette-using population in China. The snowball sampling technique employed in this study may have introduced systematic selection bias, particularly regarding gender stratification patterns. A predominantly male-dominated initial participant pool could propagate through referral chains, potentially affecting demographic representativeness. Furthermore, it should be noted that our sampling methods may not be able to reach some groups which also has an impact on the representativeness of the demographics, including Covert users – individuals who primarily use e-cigarettes in private settings (e.g., home-based users), older adult populations with limited social engagement, or those with reduced social connectivity; Subjects vulnerable to social desirability bias – particularly female users who may underreport usage frequency or modify stated motivations due to perceived social stigma surrounding vaping. Fifth, the potential e-cigarette dependence scale used in this study has not undergone further reliability and validity testing in the Chinese. Although we conducted a preliminary Alpha coefficient test of our findings, yielding an Alpha coefficient of 0.862, and we followed the guidance provided by the Diagnostic and Statistical Manual of Mental Disorders Fifth Edition for assessing dependence addiction, the accuracy of the research results may inevitably be affected to some extent. Despite these limitations, the results of the study highlight important differences in e-cigarette use groups by gender.

## 5 Conclusion

Among Chinese e-cigarette users, females preferred e-cigarettes with special flavors and without or with moderate concentrations of nicotine. The gateway effect was more prominent in females and adolescents, and the induction effect was more significant in males. There was no significant correlation between addiction dependence on e-cigarettes and gender. “To quit using traditional cigarettes and to like the scent of e-cigarettes” were prominent features of males’ motivation, while “self-perception” was prominent for females’ motivation.

## Data Availability

The raw data supporting the conclusions of this article will be made available by the authors, without undue reservation.
